# Fiordilatte Cheese Fortified with Inulin from *Cichorium intybus* or *Cynara cardunculus*

**DOI:** 10.3390/foods10061215

**Published:** 2021-05-27

**Authors:** Maria Grazia Melilli, Cristina Costa, Annalisa Lucera, Lucia Padalino, Matteo Alessandro Del Nobile, Amalia Conte

**Affiliations:** 1 National Council of Research, Institute of BioEconomy, Via Paolo Gaifami 18, 95126 Catania, Italy; mariagrazia.melilli@cnr.it; 2 Department of Agricultural Sciences, Food and Environment, University of Foggia, Via Napoli, 71121 Foggia, Italy; cristina.costa@unifg.it (C.C.); annalisa.lucera@unifg.it (A.L.); lucia.padalino@unifg.it (L.P.); amalia.conte@unifg.it (A.C.)

**Keywords:** inulin, fiordilatte cheese, fiber, fortified food

## Abstract

The influence of two different types of inulin added to fiordilatte cheese was assessed on product quality during the proper refrigerated storage period. To this aim, the fresh cheese was produced by a pilot plant, adding inulin, either from chicory (low degree of polymerization) or from cardoon (high degree of polymerization), during the stretching phase of the production process. Microbiological stability, sensory acceptability, texture and color changes of fortified dairy food during storage were measured and compared to the control cheese. Results suggest that inulin from different sources, even if characterized by a different degree of polymerization, can keep the texture and color of fiordilatte during storage. Microbiological analyses highlight that inulin seemed to promote a faster *Pseudomonas* spp. growth; however, the viable cell concentrations were found to be comparable in all the samples after one week. *Enterobacteriaceae* growth was faster when inulin from chicory was used. Sensory analysis shows that inulin addition to fiordilatte promoted the sensory quality preservation during storage; in fact, the fortified cheese overall quality was found to be always higher (*p* < 0.05) than that of the control sample, thus suggesting that inulin addition to fiordilatte represents a valid strategy for its fiber fortification.

## 1. Introduction

Inulin is a dietary fiber belonging to fructans, used as common ingredient in the food industry, for dairy and bakery products, for beverages and ice cream [1]. It is used for both technological and nutritional properties. The principal sources of inulin are asparagus root, garlic, chicory root, Jerusalem artichoke, cardoon and dahlia tubers, with an inulin content from 15 to 20% [2,3,4]. The physico–chemical properties of inulin are strictly linked to the degree of polymerization that represents the size distribution of the polysaccharide chains in the carbohydrate polymer [5]. Non-digestible oligosaccharides of long chain length are more resistant to saccharolytic fermentation and are more slowly biodegradable than compounds of shorter chain, so their fermentation will take a longer time than oligofructose fermentation, resulting in more residual carbohydrate breakdown in the distal colon [6]. Thanks to its proven nutraceutical effect, inulin could be used for novel food design [7,8].

From a technological perspective, inulin is used as a sugar replacer or as fat replacer [9]. In particular, inulin contributes to the enhancement of the mouth-feel, rheological, creaminess, textural and sensory aspects of soft low-fat cheese [5,9]. Different works have described the benefits for human health and the prebiotic effect of inulin, due to the resistance of dietary fiber during digestion [2,9]. Inulin positively controls the intestinal transit, the cholesterol and the triglycerides concentration in the blood and improves the composition of intestinal microflora [9,10]. Some researchers highlight that inulin can improve the absorption of magnesium and calcium contained in foods, thus decreasing the risk of osteoporosis [10].

In the scientific literature, the effect of inulin on several dairy products such as ice cream [11], fat-free dairy desserts [12], symbiotic Petit-Suisse cheese [13], milk beverages [14], yogurts [1,15] and imitation fresh cheese [16] has been assessed. To the best of our knowledge, no studies were found on the influence of a high degree of polymerization inulin on fresh *pasta filata* cheese, such as fiordilatte, to enhance its nutritional properties. Fiordilatte is a famous Italian cheese obtained by stretching acidified curd complemented or not by the addition of lactic acid bacteria. The curd is obtained after the coagulation of cow milk by rennet and/or coagulant enzymes. Fiordilatte shows up to 60% humidity, high fat content and maximum 2% salt [17].

Fiordilatte fortification could gain great interest because of its advantage, as is the case for many dairy products, is that it may additionally provide essential nutrients (e.g., calcium, vitamins, proteins) and the fortification with other valuable compounds is a natural way to enhance functionality. For example, Angiolillo et al. [17] developed a synbiotic fiordilatte cheese composed of an edible sodium alginate coating as a carrier of probiotic microorganisms and prebiotic fructo-oligosaccharides. To increase protein content and improve the nutritive quality of a fresh *pasta filata* cheese very similar to fiordilatte, whey protein hydrolysates were proposed by Jeewanthi et al. [18] as a source of bioactive peptides. Other studies proposed selected sources of omega-3, vitamin D and selenium to fortify mozzarella cheese [19,20,21]. From a more sustainable perspective, some researchers proposed fruit and vegetable by-products to enrich spreadable cheese [22]. Due to its composition, fiordilatte cheese is apparently a good substrate for fortification with natural compounds because nutritionally it is free of fibers [23]. On the basis of these considerations, this study aimed to produce a fortified fiordilatte, comparing inulin from chicory and inulin from cardoon plant, characterized by a different degree of polymerization.

## 2. Materials and Methods

### 2.1. Reagents and Standards

Hydrochloric acid (HCl) 3N was purchased from LabChem Inc (Zelienople, PA, USA), sodium hydroxide 50% (*w*/*w*) was purchased from J.T. Baker (Deventer, Holland), and α-glucose, fructose, and sucrose were purchased from Sigma-Aldrich (Steinhem, Germany). Deionized (DI) water, Type I reagent grade, 18 MΩ-cm resistivity, was obtained by using a Milli-Q system from Millipore (Milford, MA, USA).

### 2.2. Inulin

Inulin from chicory (*Chicory intibus*) was purchased by Beneo GmbH (Orafti^®^ HP, Mannheim, Germany). The degree of polymerization was ≥23 of fructose units (low DP). Inulin from cardoon plants (*Cynara cardunculus*) was extracted and purified at the laboratory level as reported by Sillitti et al. [24] and Padalino et al. [25]. Briefly, fresh roots (consisting of both primary and secondary roots) were washed in cold tap water, scraped and ground to a fine powder. 100 g of the original homogenate was diluted ten-fold with DI water and put in a boiling water bath for 30 min. After cooling to room temperature, the extract was filtered and centrifuged at 3000× *g* for 5 min. The inulin extracted was precipitated at 0 °C overnight. The supernatants were removed and inulin was washed with distilled water and precipitated at 0 °C overnight. The washing process was repeated until the inulin was white. After the sixth cycle of washing, solutions of inulin were injected in HPAEC PAD to follow the purity. When the baseline of chromatograms appeared clear (threshold accepted under 10 nC) and the inulin color, determined by colorimeter (Minolta CR 400), had L* values above 85, inulin was accepted for purification. Furthermore, inulin was lyophilized in sterile Petri dishes and used for fiordilatte production. The humidity in lyophilized inulin was determined in a thermo-ventilated oven at 105 °C, resulting in less than 0.5 g/100 g of fresh weight. The inulin characterization was performed as follows: 1 g of the original homogenate was diluted with water (10 mL) and put in a boiling water bath for 30 min. After cooling to room temperature, the extract was centrifuged. A part of this fraction was diluted with distilled water to analyze free sugars (glucose, fructose, and sucrose) and another fraction was hydrolyzed to determine the total fructose amount. The analyses were performed using high-performance anion exchange chromatography with pulsed amperometric detection (HPAEC PAD) (Thermofisher 3000, Sunnyvale, CA, USA), under the conditions described in Padalino et al. [25]. The degree of polymerization was determined on first fraction and the maximum DP was recorded counting the peaks over a threshold of 10 nC of the chromatograms obtained in runs of 120 min under the following elution conditions: 90 mM NaOH with 50 mM Na-acetate for 1 min followed by a linear gradient from 50 to 500 mM Na acetate in 90 mM NaOH over a 120 min period with a flow rate of 0.8 mL/min. The average chain length (mean DP) was calculated as (F − f − 0.525s)/(G − g − 0.525s), where F and G are total fructose and glucose after acid-hydrolysis and f, g, and s are the reducing free sugars fructose, glucose, and sucrose before acid hydrolysis. The mean DP of cardoon inulin resulted ≥50 fructose unit.

### 2.3. Fiordilatte Production and Packaging

Fiordilatte cheese was produced using a pilot plant, by direct acidification with lactic acid. At first, 150 L of raw milk were processed by the pilot plant (MilkyLab, Modena, Italia) located in Foggia, at the Food Technology laboratory of the University. Milk with 3.7 g L^−1^ of fat and 3.45 g L^−1^ of protein was purchased from Granarolo S.p.A. (branch office of Gioia del Colle, Italy) and transported under refrigerated conditions to the laboratory. Cold milk (7.5 ± 0.5 °C) was acidified with lactic acid solution (70:30 *v*/*v*) up to pH 5.73 ± 0.02. For the coagulation phase, the milk was heated (37.5 ± 0.2 °C) and 0.18 mL Kg^−1^ of liquid rennet (CHY-MAX^®^ Plus, CHR HANSEN, Hoersholm, Danimarca, 200 IMCU/mL) was added. Subsequently, the whey was removed and the obtained curd split into three parts. In particular, 9 kg of curd were starched with a stretching machine set at 80 °C adding 14 g Kg^−1^ of NaCl. In this step, 500 g of inulin from chicory—*Cichorium intybus* (IC) or inulin from cardoon—*Cynara cardunculus* (IS), previously solubilized in 1 L of water, were added to the curd. According to other studies reported in the literature, the incorporation of inulin was made after the draining step of curd to reduce its loss in the whey [13,26,27]. Fiordilatte cheese without inulin was also produced as a control sample (CNT). The obtained fiordilatte (50 g) was cooled in water (16 ± 1 °C) for 2 h and then four pieces of fiordilatte cheese were packaged with a brine solution (tap water) in a polypropylene tray, which was closed with a multilayer film (oriented polyamide/polypropylene 75/15 µm) on the top by using a thermo-sealing machine (Orved, Musile di Piave, Venezia, Italy). All the fiordilatte samples were stored at 4 ± 1 °C for 9 days. Duplicate batches were produced for proper repetition.

### 2.4. Texture Analysis

Fiordilatte cheese samples were uniformly sliced to a thickness of 15 mm. Cylindrical samples (28 mm diameter) were cut from the center of each cheese slice using a circular cutter. Compression tests were conducted using a Texture Analyzer Zwick/Roell model Z010 (Zwick Roell Italia S.r.l., Genova, Italy). An insert plate fixed in the universal work platform (100 × 90 × 9 mm) and compression die plate (75 mm diameter) were parallel plates inside which the cylindrical cheese samples were placed. The maximum force (F_max_) required to compress the cheese sample to a predetermined level of penetration against a rigid back plate using a cylindrical plunger was recorded for each sample tested. Pre-load of 0.3 N, load cell of 1 kN, maximum percentage deformation of 50% and constant crosshead speed of 100 mm/min were the experimental conditions. For each sample, five replicates were performed. Values of F_max_ are expressed in N.

### 2.5. Color Analysis

Color in terms of L* (lightness), a* (redness/greenness) and b* (yellowness/blueness) values was determined using a Chroma Meters CR-400 (Minolta Chromameter, Konica Minolta Business Solutions Italia Spa, Milan, Italy). The white tile was used as the standard. Measurements were taken in triplicate on the external and internal surface of fiordilatte. Results were recorded as the mean of measurements. Chroma and ∆E were also calculated at each sampling time [28].

### 2.6. Sensory Analysis

A quantitative descriptive analysis was used for the comparison of samples (UNI 10957:2003 Sensory analysis—Method to define the sensory profile of foods and beverages). Sensory evaluation was conducted by 7 trained panelists, members of the Food Packaging laboratory of the University. The panel had previous experience in testing cheese before this study; however, the panelists were retrained over 2 days (2 sections per day), to decide the appropriate descriptive terms for sensory evaluation. After retraining, the panelists evaluated product firmness, color, odor and ultimately the overall quality [23]. The analysis of the texture was performed by touching the surface of the products with fingers and evaluating the degree of surface fraying with movements from top to bottom of the surface. To judge the overall quality of cheese, the following product characteristics were also taken into account: white porcelain color, smooth surface, tight shut-off, elastic release of buttermilk after cutting, lack of holes and typical milk smell. Each taster evaluated a set of four samples each labeled with a random three-digit code. Samples were stored in the sensory analysis laboratory at room temperature before tasting and the order of presentation was different for each accepted subject, to avoid mutual interference. The tasters, using individual tasting booths in the hall of sensory analysis of the Food Packaging laboratory, were asked to judge the products by using a 7-point scale where a score equal to 4 indicated the threshold for cheese acceptability [29].

### 2.7. Microbiological Analyses and pH Determination

Fiordilatte cheese (20 g) was diluted in a sterile saline solution (0.9%) and homogenized in a blender (Stomacher, International PBI, Milan, Italy). Decimal dilutions of homogenate cheese were made in a saline solution and plated on selective media for the determination of lactic acid bacteria, lactococci, total bacterial count, *Enterobacteriaceae* and *Pseudomonas* spp. using the following media (all from Oxoid, Milan, Italy) and conditions: de Man Rogosa and Sharpe agar, supplemented with cycloheximide (0.17 g L^−1^) (Sigma, Milan, Italy) incubated under anaerobic conditions at 37 °C for 48 h for lactic acid bacteria; M17 agar, incubated at 37 °C for 48 h for lactococci; Plate Count Agar, incubated at 30 °C for 48 h for total bacterial count; Violet Red Bile Glucose Agar incubated at 37 °C for 24 h for *Enterobacteriaceae*; Pseudomonas Agar Base, added with SR103 selective supplement incubated at 25 °C for 48 h for *Pseudomonas* spp. A pH meter was used to measure the pH of fiordilatte samples (Crison, Barcelona, Spain). All the analyses were performed in duplicate on two different samples.

### 2.8. Statistical Analysis

All the experimental data were compared by one-way ANOVA analysis. A Fisher’s test, with the option of homogeneous groups (*p* < 0.05), was used to determine thesignificance differences among samples. To this aim, Statistica 7.1 for Windows (StatSoft Inc., Tulsa, OK, USA) was used. 

## 3. Results and Discussion

The main findings recorded for each quality parameter were described and discussed in detail, comparing fortified cheese with each other and with control samples.

**Figure 1:**
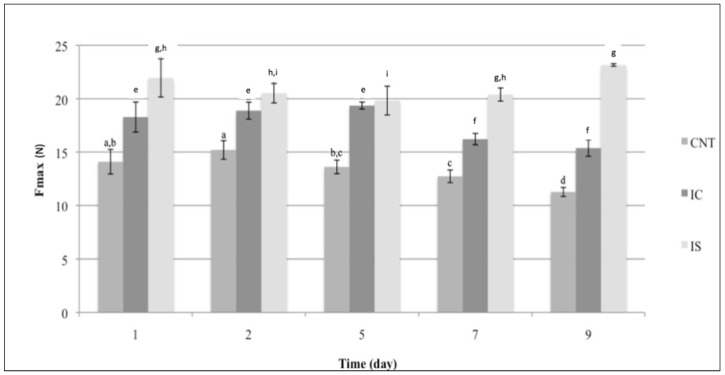
F_max_ (N) of fiordilatte cheese with inulin from chicory (IC), from cardoon (IS) and without inulin (CNT), during storage time. ^a**–**i^ Different letters indicate significant differences (*p* < 0.05) within each sample during storage time. Values are means of twenty replications ± standard deviation.

As regards texture properties, Figure 1 shows the values of F_max_ obtained for fiordilatte cheese samples. Due to the progressive product hydration [23], the measures started the day after the production and packaging to allow the equilibrium between brine and fiordilatte being established. As can be inferred from the figure, no definitive trend of the Fmax value of experimental cheese samples was observed during storage. The most striking feature of the figure is that the CNT samples recorded values of F_max_ between 10 and 15 N, whilst the IC sample recorded values of F_max_ between 15 and 20 N and the IS sample recorded values of F_max_ between 20 and 25 N. Therefore, during the entire observation period, the control cheese maintained F_max_ values lower than those found for the two fortified cheese samples. According to other studies reported in the literature, it is recognized that the physico–chemical properties of inulin tend to modify the texture of dairy products [5]. However, data recorded for samples to which inulin was added are included in the range of standard values of fiordilatte or mozzarella cheese [30,31].

It is also worth considering that the microbial spoilage and brine presence cause a loss of texture in fiordilatte during storage [23,32]. In this investigation, a marked reduction in F_max_ was recorded in the control sample; whereas cheese added with inulin better keeps the initial texture value, above all when inulin with a higher degree of polymerization was used (IS). This difference between the two inulin types can be ascribed to their different degree of polymerization and to the interaction of inulin with water. Tungland and Meyer [33] highlight that the inulin source does not directly influence the water-binding capacity, but it determines the length, particle size and porosity of inulin, thus affecting its physico–chemical properties. Other factors such as pH, ionic strength, concentration of inulin and interaction with other water-binding ingredients could also influence textural changes during storage [5].

**Figure 2:**
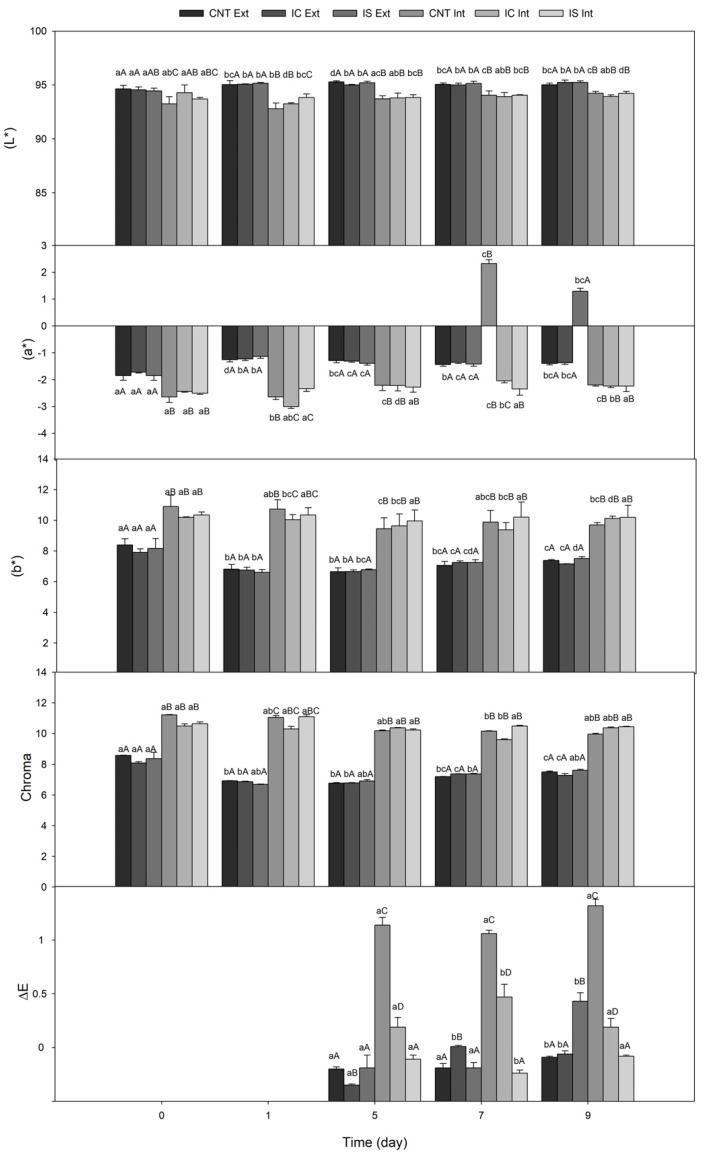
Color parameters (L*, a*, b*, Chroma and ∆E) for external (Ext) and internal (Int) surface of cheese with and without inulin. CNT: control; IC: fiordilatte with inulin from chicory; IS: fiordilatte with inulin from cardoon. Values are means of four replications ± standard deviation. ^a–d^ Statistical differences at *p* < 0.05 among storage times within the same sample; ^A–D^ Statistical differences at *p* < 0.05 among samples within the same storage time.

In Figure 2, L*, a* and b* values together with Chroma and ∆E were reported for all fiordilatte cheese samples (CNT, IC and IS). Data suggest that during the entire storage period, cheese with inulin performed similarly to the control sample, thus confirming that the selected fiber, regardless of its polymerization degree, did not cause any detectable color change to fiordilatte [9].

As long as the color space parameters are concerned, in each sample, some changes occurred during storage, both in internal and external parts of fiordilatte samples. These changes can be ascribed to the brine [23,34] and to the natural physico–chemical spoilage [35], which brings about variations in light values (L*) and shift on yellow (b* positive) and green (a* negative) axes. In particular, a decrease in the external Chroma values was observed during storage, thus indicating a modification in the color brightness. Small oscillations of the internal Chroma values were also recorded. In terms of Chroma values, differences among cheeses with and without inulin were not significant (*p* < 0.05).

∆E indicates the distance in three-dimensional space between the initial L*, a* and b* values. In our study, color changes during storage were observed for both internal and external surfaces of the CNT sample. On the other hand, the addition of inulin reduced color changes over time, above all in the internal surface. Among the two inulin types, the lowest internal ∆E values were observed for the sample with inulin from cardoon.

**Figure 3:**
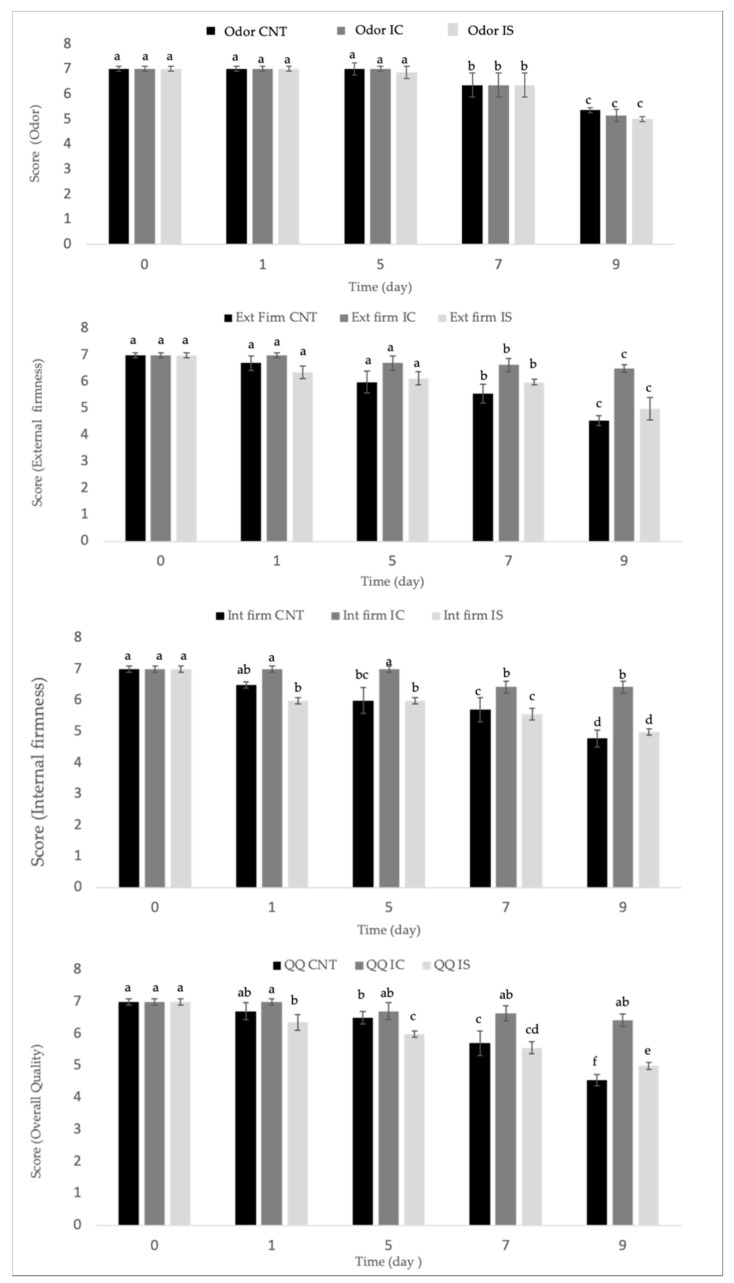
Odor, firmness and overall quality of fiordilatte. Values are means of four replications ± standard deviation. CNT: control; IC: fiordilatte with inulin from chicory; IS: fiordilatte with inulin from cardoon. Values are means ± standard deviation. ^a–f^: means of each parameter with different letters are significantly different (*p* < 0.05).

In Figure 3, the evolution during the refrigerated storage of the fiordilatte sensory quality as determined by the panel test was reported. Odor, external and internal firmness, together with overall quality are reported. Data on the color are not reported because for this specific attribute, the samples were found all to be equal over time, with scores accounting for 7 for the entire observation period. Therefore, differences recorded from the instrumental color measurement were not revealed by the panel test, who recognized for 9 days in all the sample a good white porcelain color and a smooth cheese surface.

It is worth noting from data reported in the figure that the addition of inulin did not greatly affect the sensory quality of fiordilatte cheese; in fact, comparable scores were recorded between the control and the fortified cheese samples. This result is in accordance with other studies dealing with application of inulin to fresh cheese, that also found a general product acceptance after inulin addition [9,36].

Data in the figure also highlight that, over time, some changes occurred in terms of odor. This finding is not surprising because, according to the literature, the progressive odor alteration is due to the natural spoiling phenomena occurring in dairy products during storage [32,37]. It is worth noting that no differences appear among cheese with and without inulin.

As regards the internal and external firmness of fiordilatte cheese, evident changes were recorded over time. In particular, coherently with what was found in terms of instrumental texture, the panelists observed a progressive softening of the CNT sample, which became unacceptable after 9 days of storage. In contrast, the tasters perceived as acceptable for the entire observation period the internal and external firmness of fiordilatte added with both types of inulin. Considering the trend of sensory attributes, it can be inferred that internal and external firmness are the main responsible factors for differences in fiordilatte overall quality. In fact, as can be observed from data reported in the last part of Figure 3, the IC fiordilatte sample obtained the best results in terms of overall quality between the two tested inulin types. The effect of chicory inulin on the sensory quality could be ascribed to its specific degree of polymerization [9].

**Figure 4:**
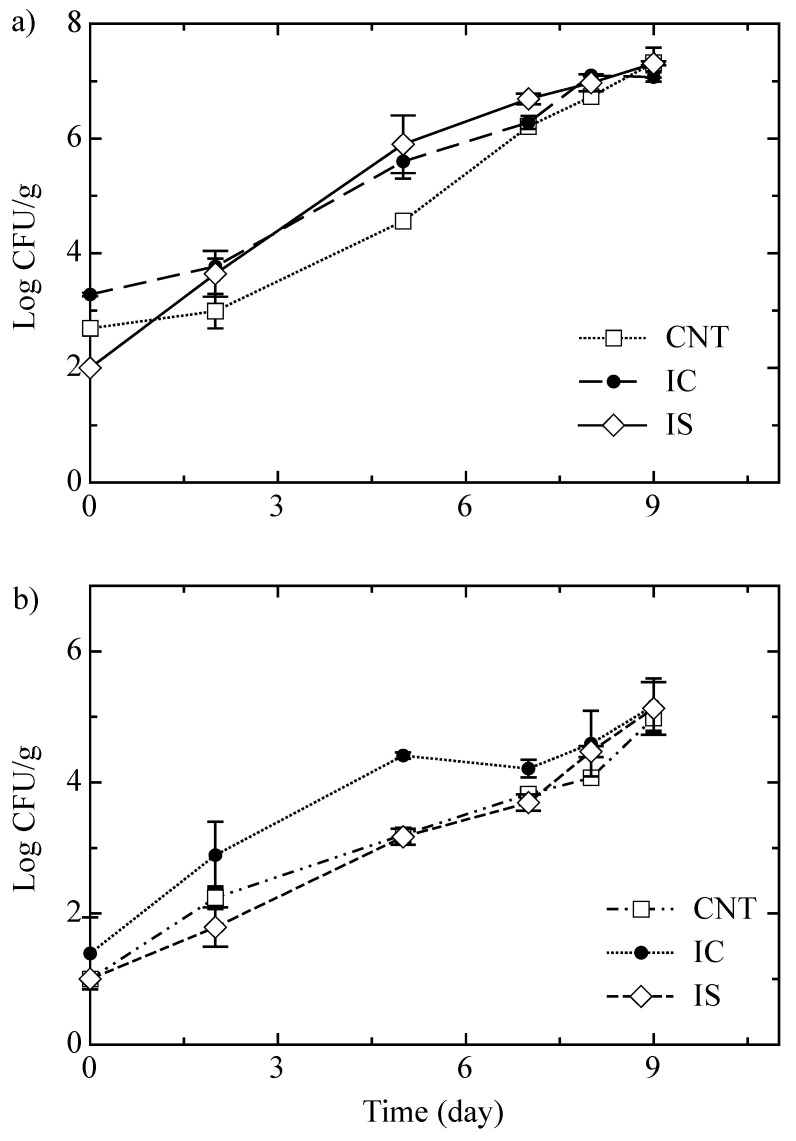
Growth curves of *Pseudomonas* spp. (**a**) and *Enterobacteriaceae* (**b**) in fiordilatte cheese with inulin from chicory (IC), inulin from cardoon (IS) and without inulin (CNT). Values are means of four replications ± standard deviation.

Regarding the effect of inulin on the microbiological quality of the investigated cheese, Figure 4a,b show the growth of spoilage bacteria (i.e., *Pseudomonas* spp. and *Enterobacteriaceae*) in all cheese samples during storage time. A progressive growth of *Pseudomonas* spp. was observed after cheese packaging in all fiordilatte samples. Specifically, up to 6 days of storage, a higher microbial concentration was found in fortified cheese compared to the control sample.

This finding can be ascribed to the ability of microorganisms to produce inulinase, an enzyme able to degrade inulin in available sugar [38]. After 6 days, the viable cell concentration was similar in all the samples (around 7 log CFU g^−1^). A similar trend was also observed for mesophylic bacteria (data not shown). Regarding *Enterobacteriaceae*, a rapid and more pronounced growth was observed in the IC sample, compared to the IS and control cheese, the viable cell count of these two latter remained comparable during the entire period of observation. Between 7 and 9 days, all the samples showed similar microbial growth, accounting for 4–5 log CFU g^−1^. A progressive increase in lactobacilli and lactococci viable cell concentration (data not shown) was recorded in all the investigated samples, without statistically significant differences among them (from 2.29 ± 0.21 and 3.08 ± 0.21 log CFU g^−1^ to about 5 and 6 log CFU g^−1^, for lactobacilli and lactococci, respectively). In terms of pH, no relevant differences were observed among samples (data not shown). For all fiordilatte cheese, small changes from 6.25 ± 0.01 to 6.35 ± 0.03 were observed.

## 4. Conclusions

The obtained results suggest that the addition of inulin, regardless of the degree of polymerization, improved the texture characteristics of fiordilatte cheese during refrigerated storage without affecting the odor and color of cheese. Quality parameters measured by instrumental tools were confirmed by the data of sensory quality as perceived by human judges. As far as the microbiological quality is concerned, even though a greater viable cell concentration was observed in the fortified cheese compared to the control sample, in the first days of storage, after about one week, the microbial concentrations were similar in all the investigated samples. Slight differences in product quality between the two types of inulin addition were found. In summary, inulin, from either cardoon plants (IS) or from chicory (IC), improved the texture properties of fiordilatte and reduced internal color changes, thus enhancing the sensory acceptability of cheese during storage. Therefore, the fortification of fiordilatte with natural fiber as inulin can enhance product quality and cheese preservation.

## Data Availability

The raw data will be made available upon request.
